# Two-Dimensional Laser-Align Device for Ultrasound-Guided Injection

**DOI:** 10.3390/jcm8071048

**Published:** 2019-07-18

**Authors:** Eric Chiwei Shiao, Po-Ling Kuo

**Affiliations:** 1 Graduate Institute of Biomedical Electronics and Bioinformatics, National Taiwan University, Taipei 10617, Taiwan; 2 Department of Electrical Engineering, Department of Physical Medicine and Rehabilitation, College of Medicine, Health Science and Wellness Center, National Taiwan University, Taipei 10617, Taiwan

**Keywords:** ultrasound-guided injection, laser assisted, long-axis injection

## Abstract

Ultrasound-guided injection is a widely used technique, however, it takes substantial amounts of time for novices to master the skill. The most critical issue to improve the accuracy of the injection is to align the needle with the scan plane of the ultrasound beam and orient the needle angle after piercing skin to aim at the targeted tissue. In the present study, we developed a two-dimensional laser align device to assist physicians to accurately position the needle in the scan plane and advance it at an angle correctly pointing to the target. The device is inexpensive, light-weighted, and easy to fabricate and accommodate for any types of ultrasound probe. Statistical analysis revealed that the assistance with our device significantly reduced the successful targeting time and times of retargeting in comparison with the traditional freehand approach or only with in-plane assistance for inexperience subjects. Our results indicate that the device exhibits great potential in effectively reducing the learning time to master the skill and speeding up the procedure for ultrasound-guided injection.

## 1. Introduction

As point-of-care has gained increasing attention, ultrasound-guided injection becomes an important and popular technique for physician. The real-time feature of ultrasonography allows clinicians to target tissue for treatment more accurately and efficiently. However, it takes substantial amounts of time for novices to master the skill. For example, the in-plane approach requires well-trained coordination between hand and eyes to accurately position the needle in the scan plane and advance it at an angle correctly pointing to the target. It is of great importance to develop tools that assist the novices in accessing the target more easily and hence to improve their learning curve.

Several devices have been developed to assist clinicians in aiming the target more quickly and precisely [1]. However, limitations still exist. For examples, the Infiniti Plus^TM^ needle guidance system (CIVCO Medical Solutions, Kalona, IA, USA) lacks the flexibility for direct adjustment of needle angle once the needle is inserted into the tissue [2]; the electromagnetic tracking system [3], optical tracking system [4,5], and robotic assistance system [6] are too large and expensive to be used in regular clinics. In contrast, laser assisting approaches are relatively inexpensive and compact-sized. However, most of the recently developed laser-based systems for needle guidance [7,8,9] only focus on the assistance in positioning the needle in the scan plane, without aiding its insertion angle. 

In this research, we developed a novel two-dimensional (2D) laser-align (LA) device to help clinicians position the needle in the scan plane and at the right angle to precisely access the targeted tissue in ultrasound-guided injection. The device is inexpensive, light-weighted, and easy to fabricate and accommodate for any types of transducer. The device is switchable among three modes: 2DLA assisting, one-dimensional (1D) in-plane LA assisting without guidance for the inserting angle of the needle, and no LA assisting, namely the traditional freehand approach. We further compared the changes in the performance of ultrasound-guided targeting for novices and experienced physicians when assisted by different modes.

## 2. Materials and Methods

### 2.1. Device Fabrication

The 2DLA device consisted of a 5 V power module containing two coin batteries, a laser module, a clip, and a transparent film printed with slanting lines corresponding to various angles for needle advancement. The laser module included two laser units and on-off and brightness were independently controlled by two sets of switch and variable resistor, respectively. This design allowed users to switch the device function among the 2D, 1D, or no LA assisting mode. The variable resistors let the user change the brightness of lasers in an environment of various darkness. 

The 2DLA assistance was accomplished by composing the laser module with two miniature laser diodes and their associated lenses, such that a laser line and a laser point were projected along and on a line aligned with the scan plane of the ultrasound beam, respectively. The pointing laser unit was used to guide the needle angle when entering the tissue. This was done by matching the projected laser point with a particular mark on the surface of the syringe body, while the marks corresponding to various needle angle were determined using the method described below. The lining laser unit was converted from a pointing laser unit with a half cylinder shaped lens, which refracted a point light source into a line one. It emitted a sector plane which aligned with the scan plane of the ultrasound beam. The sector plane projected a line on the skin surface next to the transducer and the point where the line began indicated the point where the needle tip was expected to enter the skin.

**Figure 1:**
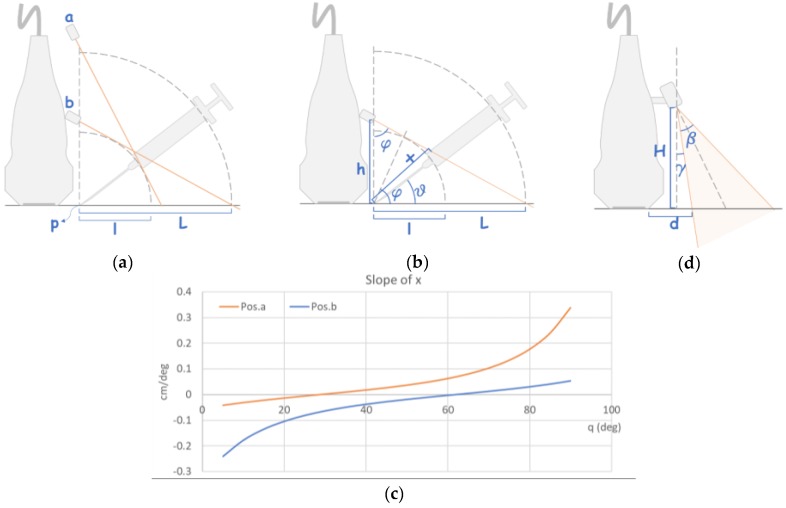
Geometric representation of the proposed positioning of the laser units. (**a**) Proposed setups of the pointing laser unit. (**b**) Geometric characteristics of the setup. (**c**) Profiles of slope of *x* against various needle angles. (**d**) Geometric layout of the lining laser unit.

The clip was custom-made by 3D printing to fit a 10 MHz ultrasonic probe (BenQ, Taipei, Taiwan). It firmly mounted the laser units onto the probe at a specific angle, such that the pointing laser always projected on the syringe surface whatever the needle angle was and provided sharp laser contrast for needle and syringe positioning. The angle was determined as follows. Given a syringe of body length *L* and its attached needle of length *l*, the syringe body should be confined within the region between the interior and exterior of two concentric circles of radius *L* and *l*, respectively, as the needle angle to the skin changes. It is assumed that the circles are centered at point *p*, where the needle tip was placed, as shown in Figure 1a. To ensure that the spot emitted from the pointing laser unit always projects on the syringe body as the needle angle changes, the unit could be positioned either at position *a* or *b* as shown in Figure 1a, both of which are close to the two ends of the syringe body when the needle angle was 90° to the skin, respectively, and the laser emitted from both positions is tangent to the inner circle. These setups allowed the projection to cover the whole syringe body and hence provided better spatial resolution for angle guidance.

Let the angle of the laser light with respect to the line vertical to the skin, and that of the needle to the skin be *φ* and *θ*, respectively, and the distance from the needle tip to the point where the laser light projected on the syringe body be *x* (Figure 1b), we have

(1) $$\varphi_{a}=\sin^{-1}\left(l/\bar{pa}\right)=\sin^{-1}\left(l/L\right),$$

(2) $$\varphi_{b}=\cos^{-1}\left(l/L\right),$$

(3) $$\bar{pb}=L\times \cot\varphi =l\times L/\sqrt{\left(L^{2}-l^{2}\right)},$$

(4) x=h(sinθ+tan(φ−θ)cosθ)=hsinφsec(φ−θ),

(5) $$x^{\prime }\equiv dx/d\theta =-h\times \sin\varphi \tan\left(\varphi -\theta \right)\sec\left(\varphi -\theta \right),$$

where *φ*_a_ and *φ*_b_ represents the angle *φ* when the unit was set at position *a* and *b*, respectively, *h* denotes $\bar{pa}$ or $\bar{pb}$ in each condition, and *x*’ is the slope of *x* against various *θ*.

Figure 1c depicts the change in *x*’ as the needle angle varied. Note that larger *x*’ offered finer spatial resolution for the guided points projected on the syringe body. Thus, the laser emitted from position *a* were projected on a wider range at larger needle angle, while that of position *b* was opposite. Since the needle angle is usually smaller than 45° in regular clinical practice, we chose position *b* to setup the pointing laser unit. The projected range of the pointing laser calculated using Equation (2) and Equation (3) would be the widest as the needle angle varied from 0° to 30°, if *φ* and $\bar{pb}$ were set to be 61.5° and 5.42 cm, respectively. An array of marks corresponding to various *θ* was determined using Equation (4) and labeled on the syringe. Thus, the needle was expected to enter the skin at a specific angle if the emitted laser spot was aligned with the mark corresponding to the angle.

The lining laser unit was set close to the pointing one to save the device size. Figure 1d demonstrates the geometric layout of the setup. The emitting angle of the laser sector plane is denoted by *β*, while the plane was aligned with the scan plane of the ultrasound beam and projected as a line on the surface of the skin or the syringe. The vertical distance between the unit and the skin surface is *H*, and *d* represents the distance between the end of the probe and the beginning of the projected line, which corresponds to point *p* in Figure 1a. Note that *d* is usually about 1 cm in clinical setting. Hence, the unit had to be tilted and *γ* represents the angle between the vertical line through the emitting point of the laser and the boundary of the sector plane, and we have

(6) $$\gamma =\tan^{-1}\left(d/H\right).$$

Note that the unit was tilted by an angle of *γ* + 0.5*β*. We set *β* and *γ* as 52.7° and 9.46°, respectively, and *H* = 6 cm, such that the two units were separated by 0.58 cm to reduce the device size.

**Figure 2:**
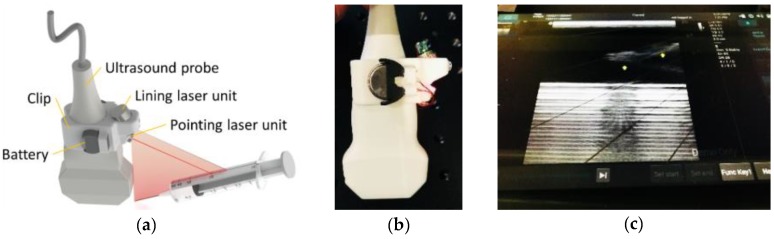
Schematic and photographs of the 2DLA device. (**a**) Diagram simulating the device in work. (**b**) A device assembled with an ultrasound probe. (**c**) The transparent film printed with the slanting lines for various needle entering angle and attached to an ultrasound screen.

**Figure 3:**
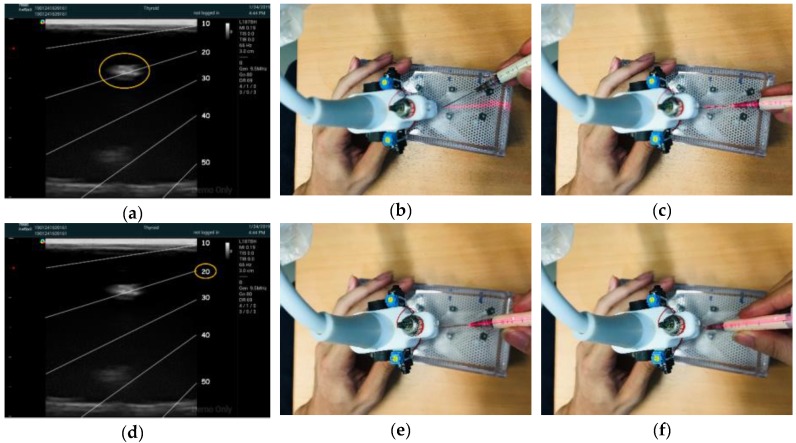
Procedure of needle entering guided with the 2DLA mode. (**a**) Position the probe to clearly visualize the target (highlighted by the yellow circle) on the screen. (**b**) Turn on the lasers and place the needle tip right at the beginning point of the projected laser line (the end of the laser line close to the probe). (**c**) Reorient the needle to match the projected laser line with the straight line attached on the syringe surface, which indicates the long axis of the needle. (**d**) Determine the insertion angle (highlighted by the yellow circle) using the slanting lines attached on the screen. (**e**) Adjust the entering angle to match the laser spot projected by the pointing laser unit with the mark labeled on the syringe surface corresponding to the insertion angle determined in (**d**). (**f**) Enter the needle into the phantom with persistent needle orientation until the needle tip touches the target on the screen.

A transparent film printed with slanting lines was attached to the ultrasound monitor. Each line represented a particular angle for needle entering and had the corresponding mark labeled on the syringe. The labels included marks for various angles and a line aligned with the long axis of the syringe. One was expected to enter the needle in the scan plane at correct angle to the target; once the syringe was aligned with the projected laser line and the pointing laser was pointed to the right mark that matching the angle determined by the slanting lines. Figure 2 illustrates the device prototype and the typical procedure of needle entering with the 2DLA assistance was demonstrated in Figure 3.

### 2.2. Device Performance

To evaluate the effectiveness of our device, twenty subjects were enrolled to perform ultrasound-guided targeting in phantoms. Six of the subjects were physicians experienced in ultrasound-guided injection and the other fourteen subjects had little experience in ultrasound imaging before this study.

**Figure 4:**
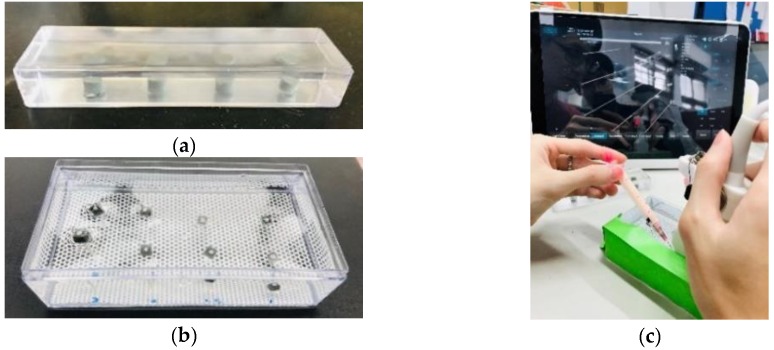
Photographs of the two types of phantom and its use in the performance test. Phantoms for (**a**) practicing, (**b**) performance test for ultrasound-guided targeting and (**c**) A subject performed ultrasound-guided targeting with a masked phantom.

The phantoms were mainly composed of liquid paraffin and thermoplastic styrene-butadiene rubber which allowed the phantoms being formed and reshaped easily. The thermal-cured phantoms were transparent, excellent for ultrasound transmission, and possessed elasticity similar to that of soft tissues. The transparency of the phantoms allowed direct visualization of the needle advancement during practice and the performance test. Blocks made of poly (dimethylsiloxane) and iron powders with high echogenicity were embedded in the phantoms to simulate targets. There were two types of phantoms used in this study (Figure 4a,b); one was for practicing and had targets embedded at the same distance to the phantom surface; and the other was made for the performance test and the distance of the simulated targets to the phantom surface varied from 1 to 2 cm. 

The performance test was conducted by asking all participants to needle three targets of different depths and each needling was guided with one of the three assisting modes in randomized order. The order of the target depths was randomized as well and the variation of target depth was to prevent subjects from being familiar with the target depth from the preceding needling. In the 2DLA mode, both the lining and pointing lasers were turned on; the subject was instructed to locate the target for injection on the B-mode image, place the needle tip at the beginning end of the projected laser line, align the needle and overlap the long-axis line labeled on the syringe body with the laser line, estimate the needle angle to the target using the slanting lines on the transparent film, match the mark on the syringe body corresponding to the angle with the laser point, and advance the needle until the tip touched the target and complete the task. The procedure in the 1DLA condition was similar except that the pointing laser was turned off and the subjects had to estimate the needle angle by themselves, while the laser units were completely turned off in the no LA assisting, freehand (FH) condition. Before the performance test, the participants had unlimited time to practice targeting in the three modes. In the performance test, the phantom was masked on the side facing the subject to prevent direct visualization of the needle advancement by the subject, while the opposite side of the phantom remained transparent for video recording (Figure 4c). For each assigned target, the subject was allowed to withdraw the needle unlimited times to adjust the needle angle until successful targeting, which was considered as the needle tip penetrated the target with the presence of the entire inserted needle on the screen. Performance was evaluated by the total duration spent from entering the needle to the phantom to the moment when a successful targeting was achieved; the number of needle withdrawals until successful targeting; and the last duration, defined as the time spent from the last withdrawal to the successful targeting, which was equal to the total time if there was no needle withdrawal.

### 2.3. Statistics

Statistical analysis was performed using IBM SPSS Statistics 25. The differences of the three performance variables grouped with respect to different subjects, task order, and target depths were analyzed with Kruskal-Wallis one-way ANOVA test. The differences of the performance conducted in the three modes were examined using Mann-Whitney U test. Significance was set as *p* < 0.05.

## 3. Results

We first asked whether the performance of individuals of the same experience level was significantly different, and whether the performance was affected by the task order and the target depths. Table 1 summarized the statistical results of the performance data (Table A1) grouped against different subjects, task order, and target depths, while the data were categorized based on the level of experience. Given the subjects of the same experience level, it appears that there was no significant difference between the averaged total durations they spent to finish the three tasks, the number of needle withdrawals, and the last durations to achieve successful targeting. Likewise, the performance was not significantly different whether the task was the first or last conducted, or the target was positioned more superficially or deeper. Detailed data were listed in the Appendix A.

**Figure 5:**
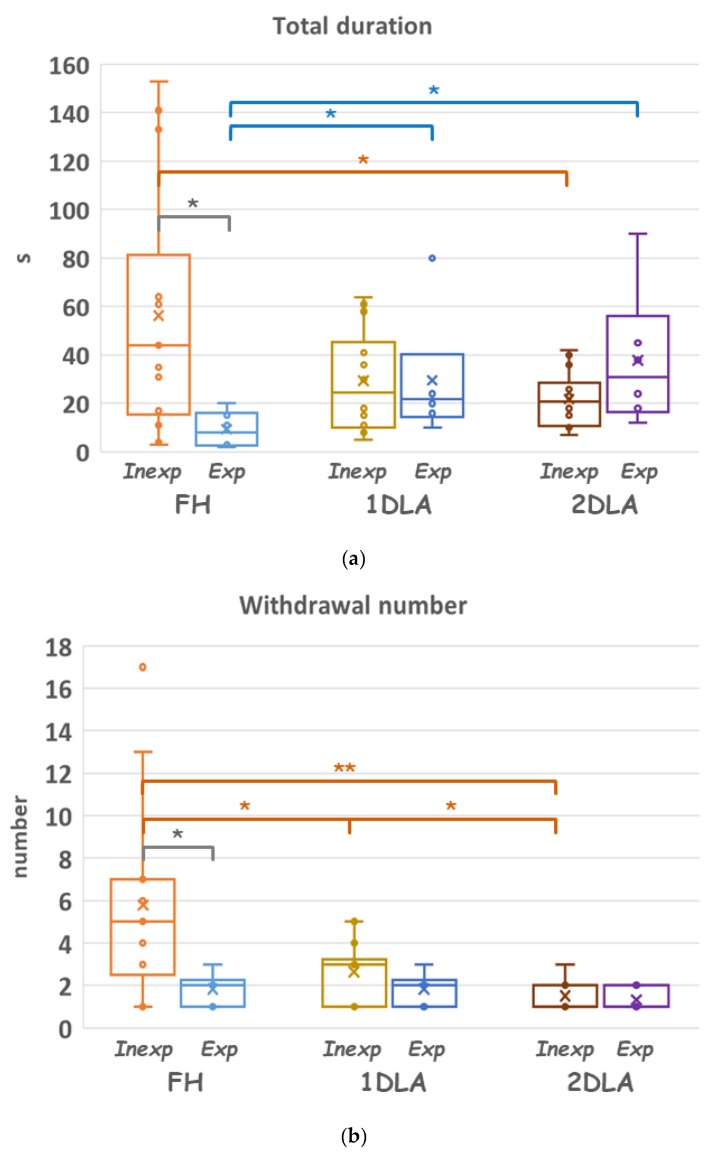

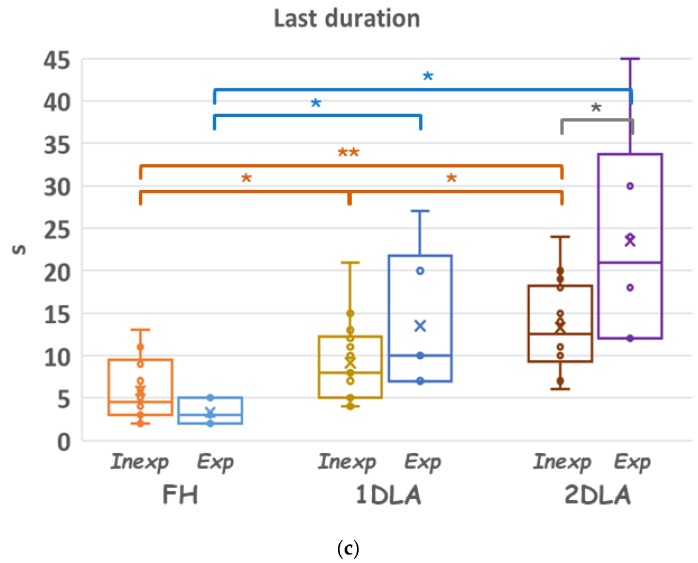
Comparisons of the performance of the subjects assisted by the three different modes. Data of (**a**) total duration, (**b**) withdrawal number, and (**c**) last duration of the inexperienced and experienced subjects performing in the freehand (FH), assisted with the lining laser (1DLA), and assisted with lining plus pointing laser (2DLA) condition. * *p* < 0.05, ** *p* < 0.01.

We next examined the performance differences in various assisting conditions. Figure 5 depicts the performance of the inexperienced and experienced subjects and the data were detailed in Table 2. As for the inexperienced subjects, they less frequently withdrew the needle for retargeting as more assistance was provided, while the last duration for successful targeting followed an opposite trend. As expected, they also spent significantly longer total duration in the FH condition than in the other two conditions. In contrast, the experienced subjects spent the shortest total duration and last duration in the FH condition than in the other two conditions. Detail *p*-values of comparisons between conditions were summarized in Table 3. As one may expect, the inexperienced subjects took significantly longer total duration and more frequent needle withdrawals than the experienced subjects in the FH condition. However, their performances were not significantly different when the LA assistances were employed, except that the experienced subjects spent significantly longer time in the last duration than the inexperienced ones in the 2DLA condition. Detail data of the two subject groups and the *p*-values of comparisons between them were summarized in Table 2.

## 4. Discussion

In the present work, we showed that for people new to ultrasound-guided injection, our novel device did help their performance in reducing the spent time and number of needle withdrawals for successful targeting. These results agree well with those reported previously [7,8,9], and we further demonstrated that our novel 2DLA design significantly helped the inexperienced operators to perform better than in the traditional freehand condition or simply assisted by a lining laser. By following the guidance provided by the pointing laser and the marks on the syringe, most of the inexperienced participants could pierce the target in one shot. This is reasonable since the 2DLA mode provided a vast amount of information for needle operation such that the subject’s expertise in ultrasound-guided injection had a minor role. This may explain the fact that most of the performance of the inexperienced and experienced subjects was not significantly distinguishable when assisted in the two LA modes. Indeed, the experienced subjects even spent much longer time to achieve the successful targeting in the two LA modes than that in the FH condition, since they already had good eye-hand coordination and individual tempo for injection and may have been confused with the laser assistance. Furthermore, it may be cumbersome to check many parameters before needle advancement in the 2DLA mode, which may explain why it took longer time for the last successful targeting in both inexperienced and experienced groups. However, we believe this drawback can be overcome as the operators become familiar with the device. Nevertheless, this mode still improved the efficacy of the inexperienced operators as the total time was less, owing to the much fewer withdrawals. The results shown in Table 1 indicate that the randomly assigned targets of different depths were successful to prevent the participants from predicting the needle angle using the experience learned in preceding tests. Furthermore, these results suggest that the task difficulty perceived by the participants was not dependent on the target depth, nor the task order. Collectively, our results indicate that the 2DLA device has a high potential to speed up the learning curve of novices in ultrasound-guided injection.

With 5.5 × 5.0 × 2.8 cm in size, 32 g in weight, and a price of about $20, our 2DLA device was compact, lightweight, inexpensive, easy to mount onto the probe, and provided immediate guidance if the operators need to change the target suddenly. Unlike the device designed by Daehee et al. [10], there is no direct contact between our device and patients’ skin or needle, which reduced the risk of infection. These features render our device competitive in clinical use or medial training when compared with commercially available products. For example, although the Infiniti Plus^TM^ needle guidance system provided by the CIVCO company has very affordable price (~$10 for each set), it prohibits repeated adjustment of the needle orientation for injection of multiple targets, or immediate replacement of syringe of various size in conditions such as the combined operation of ultrasound-guided aspiration and injection [11]. The eTRAX needle tip tracking guidance system, which is also from the CIVCO, embeds an electromagnetic sensor in the needle tip to provide needle tracking, but it costs more than 2 thousand dollars and is roughly forty times larger than ours in volume [12]. The SCENERGY system from the Clear Guide Medical attaches stereocameras to the ultrasound probe and integrates the ultrasound image with the CT/MRI image to improve needle navigation, but the assistive device is much larger than ours, and it requires attachment of labels on the subject’s skin for recognition, which may increase the risk of infection [13]. 

However, there are limitations in the present study. Although nonparametric tests were applied to make our results statistically stricter, the enrollment size was small. Furthermore, the marks labeled on the syringe for guidance of injection angle were calculated based on a flat skin surface, which may well approximate conditions when the radius of curvature of the skin is much larger than the probe size, such as scanning along the long axis of an arm. However, the guidance will be erroneous when entering the needle through skin surface with smaller radius of curvature, holding the ultrasound probe askew or using a curvilinear array probe. Additional calculation is required to fix the scale distortion arising in these conditions. For example, when our device is applied to a curvilinear probe, the slanting lines printed on the transparent film should be curved to match the sector image generated by the probe. Likewise, since the needle entering point is usually out of the image view when the probe is applied on a curved surface with a large curvature, such as scanning along the transverse plane of an arm, it is hard to predict the needle orientation unless the geometric condition between the probe and the entering point is given.

## 5. Conclusions

In summary, we developed a novel 2DLA device that provided guidance for needle orientation both for the image plane and the angle for entering. Statistical analysis revealed that the 2DLA mode significantly reduced the time for successful targeting and frequency of retargeting in comparison with the FH and 1DLA mode. Injecting quickly and accurately is one of the most challenging tasks during the training for ultrasound-guided injection. Our device exhibits great potential in effectively reducing the learning time to master the skill and speeding up the procedure.

## Figures and Tables

**Table 1:** Significances of differences between the means of the performance data categorized with respect to different subjects, task orders, and target depths. * *p* < 0.05.

	Group	Subjects	Orders	Target depths
**Total Duration**	Inexperienced	35.83 ± 22.18, *p* = 0.133	35.83 ± 10.90, *p* = 0.134	35.83 ± 3.71, *p* = 0.779
	Experienced	25.56 ± 16.34, *p* = 0.471	25.56 ± 5.99, *p* = 0.535	25.56 ± 8.20, *p* = 0.385
**Withdrawals**	Inexperienced	3.31 ± 1.90, *p* = 0.281	3.31 ± 0.85, *p* = 0.234	3.31 ± 0.36, *p* = 0.975
	Experienced	1.67 ± 0.43, *p* = 0.206	1.67 ± 0.36, *p* = 0.076	1.67 ± 0.24, *p* = 0.331
**Last Duration**	Inexperienced	9.43 ± 3.72, *p* = 0.120	9.43 ± 0.78, *p* = 0.582	9.43 ± 0.53, *p* = 0.883
	Experienced	13.44 ± 4.75, *p* = 0.925	13.44 ± 2.12, *p* = 0.804	13.44 ± 5.69, *p* = 0.162

**Table 2:** Means and standard deviations of inexperienced group and experienced group in FH, 1DLA, and 2DLA conditions with significance level of relation between two groups. * *p* < 0.05.

	Group	FH		1DLA		2DLA	
Total Duration	Inexperienced	56.21 ± 48.67	*p* = 0.012 *	29.36 ± 19.90	*p* = 0.968	21.93 ± 10.91	*p* = 0.207
	Experienced	9.33 ± 6.60		29.50 ± 23.24		37.83 ± 25.90	
Withdrawals	Inexperienced	5.79 ± 4.36	*p* = 0.026 *	2.64 ± 1.39	*p* = 0.274	1.50 ± 0.63	*p* = 0.718
	Experienced	1.83 ± 0.69		1.83 ± 0.69		1.33 ± 0.47	
Last Duration	Inexperienced	5.86 ± 3.60	*p* = 0.179	9.14 ± 4.73	*p* = 0.312	13.29 ± 5.23	*p* = 0.050 *
	Experienced	3.33 ± 1.25		13.50 ± 7.46		23.50 ± 11.54	

**Table 3:** Significance of difference in the performance of the inexperienced and experienced subjects compared between various assistance conditions. * *p* < 0.05, ** *p* < 0.01.

	Group	FH–1DLA	FH–2DLA	1DLA–2DLA
Total Duration	Inexperienced	*p* = 0.210	*p* = 0.039 *	*p* = 0.541
	Experienced	*p* = 0.026 *	*p* = 0.015 *	*p* = 0.485
Withdrawals	Inexperienced	*p* = 0.019 *	*p* = 0.001 **	*p* = 0.039 *
	Experienced	*p* = 1.000	*p* = 0.310	*p* = 0.310
Last Duration	Inexperienced	*p* = 0.039 *	*p* < 0.001 **	*p* = 0.044 *
	Experienced	*p* = 0.002 **	*p* = 0.002 **	*p* = 0.093

## Appendix A

**Table A1:** Data of each subject in inexperienced group and experienced group.

	Subject	Task	Order	Target Depth (cm)	Total Duration (s)	Withdraw Times (#)	Duration of Last Insertion (s)
Inexperienced Group	1	FH	I	1	64	4	7
		1DLA	II	1.5	15	1	15
		2DLA	III	2	10	1	10
	2	FH	I	2	61	7	11
		1DLA	II	1.5	58	5	13
		2DLA	III	1	42	2	14
	3	FH	III	2	31	3	9
		1DLA	II	1	8	1	8
		2DLA	I	1.5	26	2	11
	4	FH	III	1	44	7	2
		1DLA	I	2	36	3	5
		2DLA	II	1.5	23	2	6
	5	FH	II	2	17	5	3
		1DLA	I	1	19	3	4
		2DLA	III	1.5	11	1	11
	6	FH	I	1.5	35	6	3
		1DLA	III	1	18	3	5
		2DLA	II	2	7	1	7
	7	FH	II	1	46	4	6
		1DLA	III	1.5	5	1	5
		2DLA	I	2	10	1	10
	8	FH	II	1.5	3	1	3
		1DLA	I	2	11	1	11
		2DLA	III	1	19	1	19
	9	FH	III	1.5	4	1	4
		1DLA	II	2	37	3	7
		2DLA	I	1	15	1	15
	10	FH	II	1	11	1	11
		1DLA	III	2	8	1	8
		2DLA	I	1.5	40	2	20
	11	FH	III	1.5	133	13	5
		1DLA	I	1	64	5	21
		2DLA	II	2	24	1	24
	12	FH	I	2	141	17	2
		1DLA	III	1.5	30	4	4
		2DLA	II	1	36	3	7
	13	FH	I	2	153	7	13
		1DLA	II	1.5	61	3	12
		2DLA	III	1	18	1	18
	14	FH	III	1	44	5	3
		1DLA	I	1.5	41	3	10
		2DLA	II	2	26	2	14
Experienced Group	1	FH	III	1.5	11	2	3
		1DLA	II	2	80	3	20
		2DLA	I	1	90	2	30
	2	FH	I	1.5	15	2	5
		1DLA	II	1	24	2	10
		2DLA	III	2	12	1	12
	3	FH	II	2	20	3	5
		1DLA	III	1.5	16	2	7
		2DLA	I	1	45	1	45
	4	FH	I	1	3	1	3
		1DLA	II	1.5	20	2	7
		2DLA	III	2	38	2	12
	5	FH	II	2	2	1	2
		1DLA	I	1	10	1	10
		2DLA	III	1.5	18	1	18
	6	FH	III	1.5	5	2	2
		1DLA	II	1	27	1	27
		2DLA	I	2	24	1	24
